# Highly Sensitive Optical Fiber Pb^2+^ Concentration Sensor Based on HEMA/AM/SA Interpenetrating Polymer Network (IPN) Hydrogel

**DOI:** 10.3390/gels11100766

**Published:** 2025-09-23

**Authors:** Ning Wang, Ming He, Longjiao Wang, Chuanjie Lei, Linyufan Xiao, Yingjie Li, Shuan Liu

**Affiliations:** College of Science, China University of Petroleum (East China), No.66 Changjiang West Road, Qingdao 266580, China; z23090020@s.upc.edu.cn (M.H.); s23090002@s.upc.edu.cn (L.W.); s23090001@s.upc.edu.cn (C.L.); z24090012@s.upc.edu.cn (L.X.); 13573286524@163.com (Y.L.); ls15853492661@126.com (S.L.)

**Keywords:** IPN hydrogel, Pb^2+^ detection, optical fiber sensor

## Abstract

An optical fiber sensor based on a HEMA/AM/SA interpenetrating polymer network (IPN) hydrogel is proposed for monitoring the concentration of Pb^2+^. The Fabry–Perot interference cavity is constructed from a single-mode fiber, a ceramic ferrule, and an IPN hydrogel layer. P (HEMA co AM)/SA IPN hydrogel films were prepared by a step-by-step crosslinking method, which had good mechanical properties, swelling properties, and Pb^2+^ adsorption capacity. The Pb^2+^ concentration changes cause the interference spectrum shift of the sensor. By monitoring the wavelength shift under different Pb^2+^ concentrations, the sensor sensitivity in the range of 0~1 ppm Pb^2+^ concentration in solution is 5.0743 nm/ppm with 0.994 linearity. The influence of different proportions of IPN hydrogel on the performance of the sensor was studied. In the range of 10–90% HEMA, higher sensitivity is obtained by a small weight ratio of HEMA/AM. The sensor stability, repeatability, selectivity, dynamic response, and temperature response are also investigated in experiments. Experimental results demonstrate that the proposed sensor exhibits good stability, sensitivity, repeatability, and selectivity. Owing to its compact structure, straightforward fabrication, low cost, and good sensing performance, this sensor shows strong potential for application in monitoring Pb^2+^ concentrations.

## 1. Introduction

Rapid industrialization has exacerbated environmental pollution, with heavy metal contamination emerging as a critical threat to both human health and ecological systems. Among these contaminants, as a highly toxic heavy metal ion, lead widely exists in the natural environment. Lead ions will enter the water and cause severe aquatic pollution by the discharge of lead-containing wastewater, surface runoff, and atmospheric sedimentation. This water pollution not only directly destroys the aquatic ecosystem and affects the growth and survival of animals and plants [1,2] but also poses a threat to human health. Lead ions in agricultural products irrigated by drinking water or contaminated water sources can enter the human body, causing serious damage to the nervous system, hematopoietic system, and kidney [3,4]. Long-term exposure to lead ions may also lead to intellectual disability, anemia, hypertension, and other diseases in children [5]. Consequently, developing sensitive and real-time detection methodologies for Pb^2+^ in aqueous systems is very important for assessing pollution degree, warning of health risks, protecting water resource security, and maintaining ecosystem health.

Traditional methods for detecting heavy metal ions mainly include atomic absorption spectrometry, ultraviolet–visible spectrophotometry, inductively coupled plasma mass spectrometry, and electrochemical analysis [6,7,8,9]. These methods have good sensitivity and accuracy but suffer from several limitations, such as requiring expensive instruments, complex operations, and long testing times [10]. In comparison, optical fiber sensors have many advantages, such as resistance to electromagnetic interference, small size, high sensitivity, and the ability for remote monitoring, which showed great potential in the detection of lead ions [11,12]. In 2015, R. Verma et al. [13] proposed a fiber optic sensor based on surface plasmon resonance (SPR), which used a metal/metal oxide coating modified with pyrrole and chitosan on the surface for detecting various heavy metal ions. In 2018, N. Zhong et al. [14] proposed a three-layered D-type polymer fiber optic evanescent wave (FOEW) sensor for responding to glucose and Hg^2+^ in aqueous solutions. In the same year, Tan et al. [15] proposed a long-period fiber grating (LPFG) sensor for detecting Hg^2+^, employing arc-induced technology to inscribe LPFG on single-mode fiber and coating it with PE-AuNP to react with Hg^2+^. These studies mainly utilized composite fiber optic geometries, two-dimensional materials, and composite nanomaterials (especially materials like hollow gold nanoshells) for heavy metal ion detection, often involving complex fabrication processes and high costs. Etching, polishing, or bending fiber can enhance sensor sensitivity but may also weaken the fiber’s mechanical strength, significantly affecting the sensor’s stability and reproducibility. The thermo-optic effect of gratings can impact measurement accuracy. The bonding process between the sensitive membrane and the SPR-excited metal layer is complex, increasing the sensor’s manufacturing cost.

In recent years, the integration of functional materials with optical fiber sensing platforms has significantly advanced this field. Hydrogels, in particular, have emerged as an ideal material for constructing sensitive interfaces due to their three-dimensional network structure, high water content, excellent mechanical properties, adsorption capacity, and swelling behavior. For instance, chitosan-based hydrogels, rich in amino and hydroxyl groups, exhibit strong adsorption capability toward heavy metal ions and have been widely employed in optical fiber sensing research [16,17]. Notably, Schiff base hydrogels, formed through dynamic covalent bonding via aldol condensation reactions, not only possess tunable self-healing properties and degradability but also contain imine groups (–C=N–) that serve as effective coordination sites for metal ions [18,19,20], markedly enhancing selective capture ability for heavy metal ions. However, single-network hydrogels often face challenges such as insufficient mechanical strength, limited swelling performance, and relatively low adsorption capacity [21,22].

In 2021, Abdullah et al. [23] proposed an F-P fiber optic sensor based on a chitosan coating for Pb^2+^ measurements, with −0.091 nm/ppm sensitivity for lead concentration tests ranging from 0 ppm to 30 ppm. In 2022, Li et al. [24] developed an MZI optic fiber sensor based on a smart hydrogel, constructed by splicing a single-mode fiber between two single-mode fibers, capable of measuring lead ion concentrations in the range of 0.04–0.25 ppm with 3.936 nm/ppm sensitivity. Although these studies adequately demonstrate the application potential of hydrogels in the field of optical fiber sensing, there remains considerable room for improvement in the monitoring range and sensitivity. Interpenetrating polymer network (IPN) hydrogels [25,26], formed by the mutual interpenetration of two or more polymer networks, effectively combine the advantages of each component, enabling synergistic enhancements in mechanical properties, swelling behavior, and adsorption functionality [27,28,29]. This approach offers an innovative strategy to overcome the aforementioned limitations. Nevertheless, reports on utilizing the synergistic effects of IPN hydrogels to improve the overall performance of sensors remain relatively scarce, indicating broad application prospects for IPN-based materials in the research domain of optical fiber heavy metal-ion sensing.

This paper proposes a lead ion concentration FPI optic fiber sensor based on HEMA/AM/SA interpenetrating polymer network (IPN) hydrogel. By combining the mode interference fiber-optic sensing principle [30,31] with the heavy metal ion adsorption characteristics of IPN hydrogels, this sensor is used to detect the Pb^2+^ concentration in solutions. The prepared IPN hydrogel has good mechanical properties, swelling properties, and Pb^2+^ adsorption capacity. Changes in Pb^2+^ concentration will alter the refractive index of the hydrogel and the effective cavity length of the fiber F-P cavity, which causes the interference spectrum shift. The Pb^2+^ concentration is measured by this method. This paper verifies that the sensor has good response characteristics to Pb^2+^ concentration in solution and investigates sensor performance related to some conditions, such as IPN hydrogel ratios, dynamic response, and temperature response characteristics. The results show that the sensor has high sensitivity, good linearity, and excellent repeatability and stability. Good response selectivity is also obtained by the ion imprinting method. The sensor has a simple structure, low cost, and good performance, providing a new direction for the development of low-cost, high-performance portable lead-ion concentration sensing devices.

## 2. Results and Discussion

### 2.1. Initial Spectral Analysis

**Figure 1:**
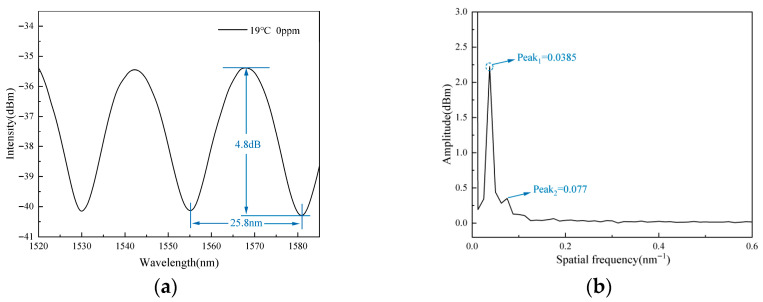
Sensor spectrum. (**a**) Interference spectrum of the fabricated sensor within the wavelength range of 1520–1585 nm. (**b**) Spatial frequency spectrum of the interference spectrum, showing two distinct peaks: a primary peak at 0.0385 nm^−1^ and a secondary peak at 0.077 nm^−1^, the latter being exactly twice the frequency of the former.

When the sensor is placed in deionized water at room temperature, the reflection spectrum of the sensor is obtained as Figure 1a showed, which has a 25.8 nm free spectral range (FSR) and a 4.88 dB contrast value. To further analyze the interference phenomenon, the interference spectrum undergoes signal demodulation processing. By the Fast Fourier Transform (FFT) method, the amplitude–frequency characteristic curve of the reflection spectrum is obtained [32,33]. Figure 1b shows the spatial frequency spectrum, where the main frequency corresponding to the FFT is 1/FSR = 0.0385 nm^−1^. The theoretical FSR value is approximately 25.97 nm, which aligns well with the measured FSR (25.8 nm) from the spectrum. In the figure, there is a prominent main peak with a secondary peak. The secondary peak is a multiple of the main peak’s frequency, indicating that the interference spectrum of the sensor is primarily determined by the interference of reflected lights at both the SMF/IPN hydrogel and IPN hydrogel/solution interfaces.

### 2.2. Sensor Response Stability

**Figure 2:**
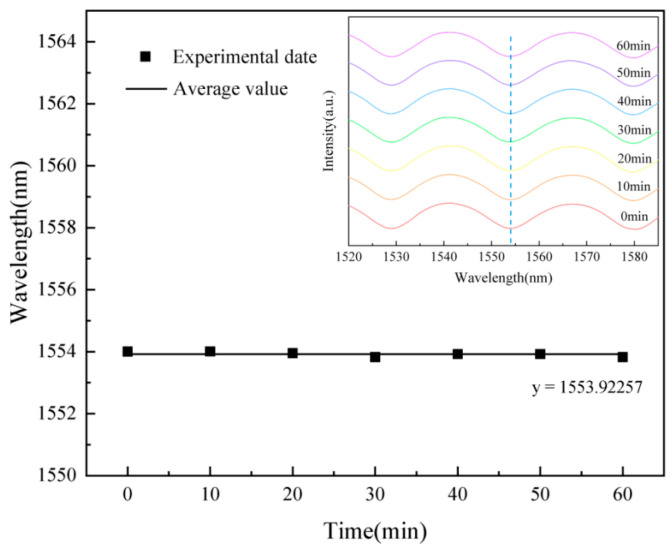
Stability response. The spectrum was recorded every 10 min to evaluate its stability; the solid line represents the mean value of the trough wavelength, and the inset displays spectra acquired at different times.

To assess the sensor’s response stability, the temperature was maintained at 19 °C. The interference spectrum was recorded every 10 min, yielding a total of seven datasets. The spectral data are displayed in Figure 2. Figure 2 shows the spectrum’s stability over 60 min, with the inset comparing spectra at different times.

It was found that the selected interferogram troughs were all near 1553.9 nm, with a relative average deviation of 0.025%. The experimental results indicate that this sensor exhibits good response stability.

### 2.3. Pb^2+^ Response Sensitivity

**Figure 3:**
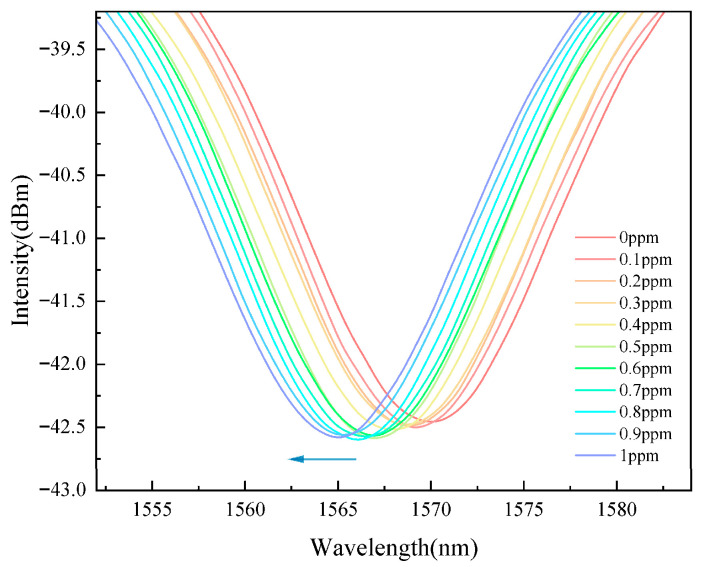
Spectral response to Pb^2+^. Response spectra of the sensor after stabilization in solutions with different Pb^2+^ concentrations; a blue shift occurs with increasing Pb^2+^ concentration.

The sensor was immersed in deionized water at 19 °C ambient temperature, and the Pb^2+^ concentration in the solution was varied from 0 to 1 ppm. The response spectrum was continuously monitored and recorded. The sensor response spectra with different concentrations of lead ions are shown in Figure 3.

It was observed that as the concentration of lead ions increased, the spectrum exhibited a blue shift.

**Figure 4:**
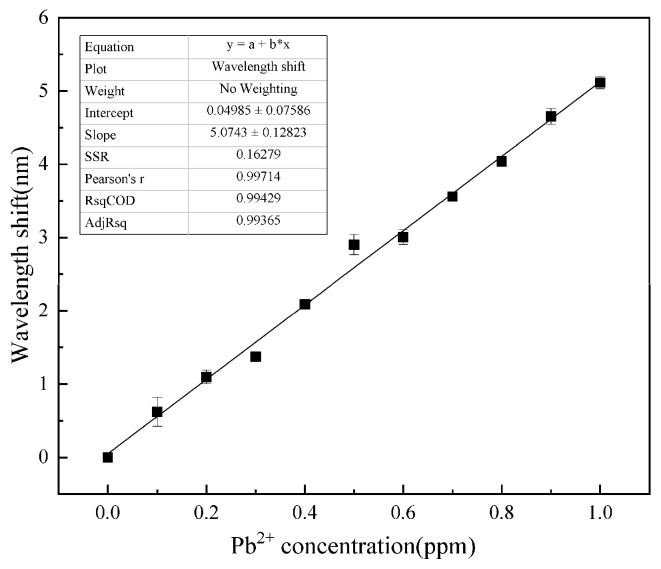
Response curve to Pb^2+^. The response sensitivity is 5.0743 nm/ppm with a linearity of 0.994.

To further illustrate the relationship between spectral wavelength shift and Pb^2+^ concentration, Figure 4 was obtained from previous spectral data. As shown in Figure 4, the sensor demonstrates a robust linear response (R^2^ = 0.994) across the 0–1 ppm concentration range, exhibiting a detection sensitivity of 5.0743 nm/ppm for Pb^2+^ ions. This underscores its high sensitivity and good linearity.

### 2.4. Dynamic Response

To investigate the dynamic response characteristics of the sensor, the spectral acquisition frequency was set to record the spectrum every 30 s during the experiment. Before altering the solution concentration, spectral data were continuously collected until a stable baseline was achieved. Under constant temperature conditions (19 °C), a quantified amount of PbCl_2_ was introduced into the solution to vary the Pb^2+^ concentration, resulting in a rapid blue shift of the interference spectrum. After each concentration change, spectral acquisition continued until the spectrum was stable enough to achieve a complete dynamic process.

**Figure 5:**
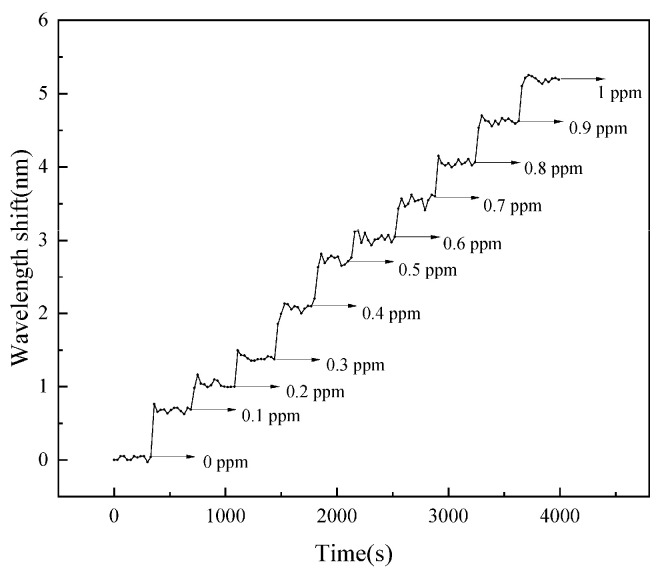
The dynamic wavelength shifts with different Pb^2+^ concentrations. The response curve exhibits a step-like ascent.

Analysis of the dynamic response spectral data revealed the relationship between the spectral shift and the response time during changes in Pb^2+^ concentration, as shown in Figure 5. It was observed that the spectral shift increased significantly with increasing Pb^2+^ concentration, exhibiting a step-like ascent in the response curve.

**Figure 6:**
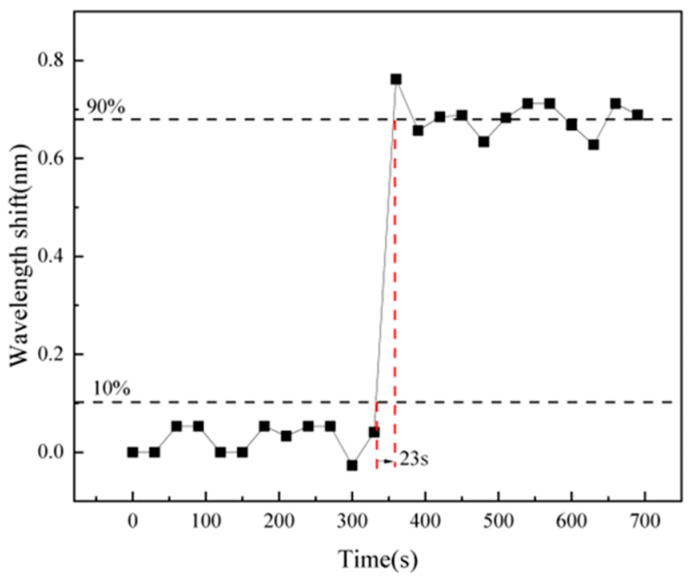
The enlarged view of the typical dynamic response result (0~700 s).

To further evaluate the dynamic response details, a magnified view of the selected region in Figure 5 is presented in Figure 6. Based on this amplified curve, the sensor’s response time, defined as the duration between 10% and 90% of the total wavelength shift, was measured to be 23 s. This behavior is primarily attributed to the adsorption characteristics of the IPN hydrogel toward metal ions. When the ion concentration increases abruptly, the hydrogel rapidly adsorbs ions, reaching approximately 80–90% of its saturation capacity within a short period. Then, the adsorption rate decreases gradually until dynamic equilibrium is attained. The results demonstrate that the sensor exhibits a favorable response speed and shows potential for practical engineering applications.

### 2.5. Sensor Reproducibility

**Figure 7:**
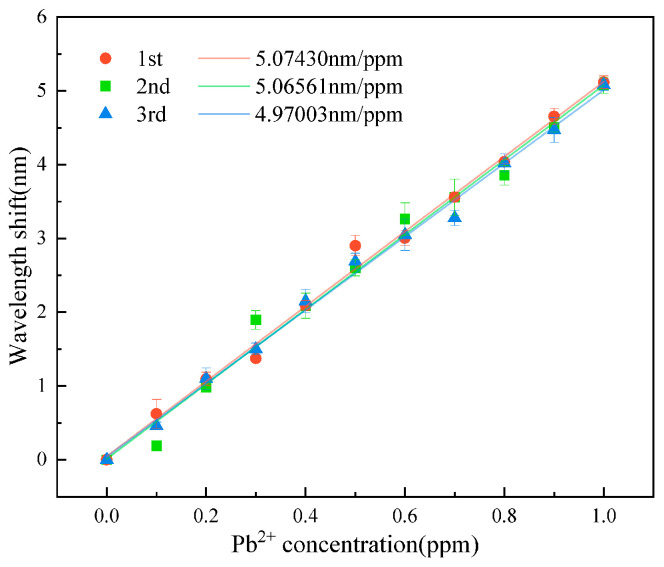
Repeated response measurements after desorption using the same sensor, showing high consistency.

In scientific research and practical applications, the sensor reproducibility is very important. Sensors with good reproducibility enhance the reliability of detection results and reduce detection costs. During our experiments, we found that immersing used sensors in deionized water effectively removes adsorbed Pb^2+^ from the sensing membrane. This desorption method is simple and does not damage the hydrogel structure. To investigate the sensor’s reproducibility, the same sensor was repeatedly measured three times, as shown in Figure 7. The three curves have a good overlap. The response sensitivities of three curves were 5.0743 nm/ppm, 5.06561 nm/ppm, and 4.97003 nm/ppm, respectively. It can be seen that the sensor exhibits excellent reproducibility.

### 2.6. Effect of HEMA/AM Ratio on Response Characteristics

**Figure 8:**
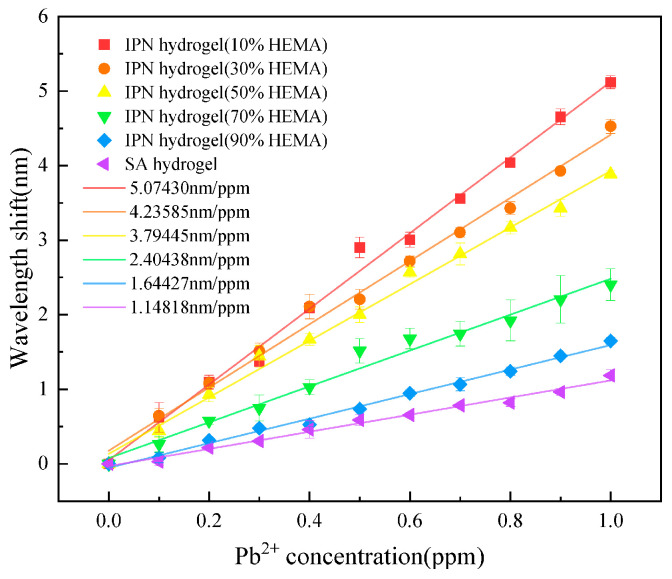
Sensitivity of sensors with different HEMA/AM weight ratios to Pb^2+^. The IPN hydrogel exhibits higher response sensitivity than the SA hydrogel, and the sensitivity increases with decreasing %HEMA.

**Figure 9:**
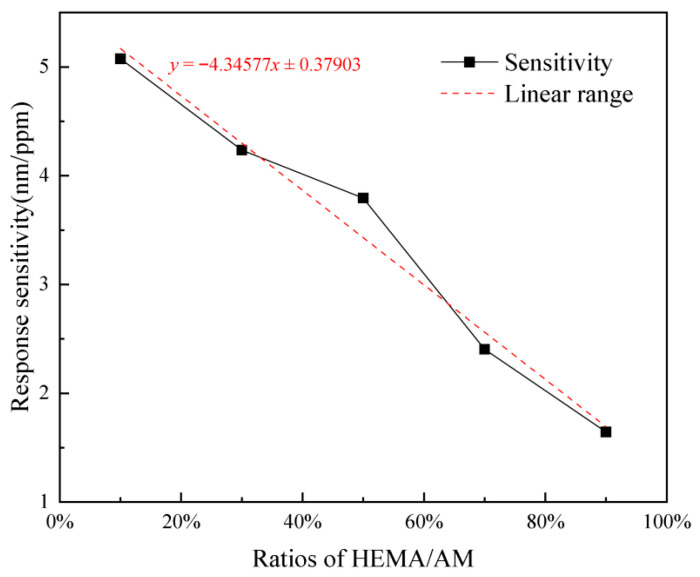
Relationship between HEMA/AM weight ratio and sensor response sensitivity. The black solid line represents the trend of response sensitivity versus the HEMA/AM ratio, and the red dashed line corresponds to the linear fitting result.

The composition of hydrogels plays a pivotal role in the adsorption of Pb^2+^ ions. Considering the composition, the effect of the HEMA/AM ratio is investigated. P(HEMA-co-AM)/SA IPN hydrogels synthesized with different mass ratios of HEMA to AM (10% ~ 90% HEMA) are listed in Table 1. We use these hydrogels to investigate the influence of different HEMA/AM ratios on response characteristics. Hence, five sensors with different hydrogel component ratios were fabricated for comparison tests. In addition, a sensor-based pure sodium alginate hydrogel with a mass fraction of 2% (prepared from 49 g deionized water and 1 g sodium alginate) was also prepared for an interesting comparison with ordinary single-network hydrogel. Finally, all six sensors were obtained, respectively, to determine their response characteristics. The initial cavity length of each sensor was approximately 35 μm. The Pb^2+^ concentration response spectra were measured by the same method as in Section 4.2, resulting in six wavelength drift curves. All curves were compiled in Figure 8. Among them, the response sensitivity of the sensor based on SA hydrogel was 1.14818 nm/ppm. While the response sensitivities of the sensors made from P(HEMA-co-AM)/SA IPN hydrogels synthesized with different mass ratios of HEMA to AM (10%~90% HEMA) were sequentially 5.0743 nm/ppm, 4.23585 nm/ppm, 3.79445 nm/ppm, 2.40438 nm/ppm, and 1.64427 nm/ppm. The response linearity of the six sensors was all above 0.98. It can be seen that compared with pure SA hydrogel, the sensors with P(HEMA-co-AM)/SA IPN hydrogels have better response sensitivity. The reduction in %HEMA can improve the Pb^2+^ response sensitivity, with the sensor exhibiting a 4.4-fold improvement at 10% HEMA concentration. This phenomenon can be attributed to the increased flexibility of the IPN at lower %HEMA [34,35], which results in more hydrophilic parts and increased active sites on the adsorbent, thereby enhancing the adsorption capacity [36,37]. Figure 9 shows the relationship between the HEMA/AM weight ratio and sensor response sensitivity. The slope of linear fitting results is −4.34577, which means that the sensor response sensitivity increases about 0.435 nm/ppm with every 10% reduction of HEMA/AM weight ratio in the range of 10–90%.

### 2.7. Selectivity Test

**Figure 10:**
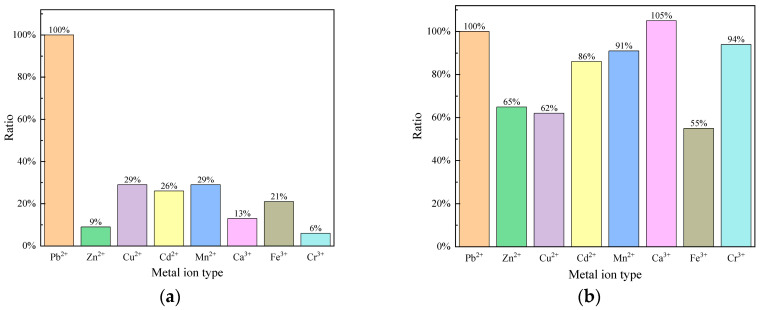
Selective measurements of sensors. (**a**) Response of the ion-imprinted sensor to different metal ions over a comparable concentration variation range. (**b**) Response of the non-imprinted sensor to different metal ions over a comparable concentration variation range.

Ion imprinting technology [38,39] was reported in recent years to affect the ion absorption ability. To enhance the sensor’s specific recognition capability for Pb^2+^, this method was introduced during the sensor fabrication process in experiments. We use saturated PbCl_2_ solution instead of CaCl_2_ solution to crosslink the hydrogel, which leaves specific recognition sites for Pb^2+^ on the hydrogel film to improve the sensor’s selectivity for Pb^2+^. To evaluate the selective response to Pb^2+^, tests were conducted in various ionic solutions, including Pb^2+^, Zn^2+^, Cu^2+^, Cd^2+^, Mn^2+^, Ca^2+^, Fe^3+^, and Cr^3+^ (the solutions of Pb^2+^, Zn^2+^, Cu^2+^, Cd^2+^, and Ca^2+^ were prepared from their chloride salts, while Mn^2+^, Fe^3+^, and Cr^3+^ were derived from their nitrate salts; all solutions were prepared using deionized water). It is worth noting that the binding of all tested metal ions to the sensor induced a blue shift in the interference spectrum, a phenomenon consistent with the contraction effect of the hydrogel during ion adsorption. The relative sensitivity ratio of the ion-imprinted sensor in different ion solutions within the same concentration range (0–1 ppm) is shown in Figure 10a. It can be evident that the sensor’s response sensitivity to Pb^2+^ is significantly higher than that to other ions. These results indicate that the sensor exhibits excellent selective response, which is highly beneficial for addressing crosstalk issues in the sensor.

For investigating the effect of the ion imprinting method, the sensor with non-ionic imprinting materials was also tested in different ion solutions within a 0–1 ppm concentration range, as illustrated in Figure 10b. It can be observed that the sensor’s selectivity for Pb^2+^ is not significantly better than that for other metal ions. Particularly noteworthy is its response to Ca^2+^, which was as high as 105%. This behavior is attributed to the non-specific adsorption of metal ions by functional groups in the non-imprinted hydrogel.

The response change to Ca^2+^ and Pb^2+^ before and after imprinting is particularly instructive. In the non-imprinted state, Ca^2+^, as the original crosslinking ion, exhibited the highest response (105%), indicating an intrinsic affinity of the network for this ion. After Pb^2+^ imprinting, the response to Ca^2+^ drastically decreased to 13%. This sharp reduction strongly demonstrates that the ion imprinting process effectively suppressed non-specific binding and successfully constructed recognition cavities highly selective for Pb^2+^. After the imprinting process, the sensor maintained a high response toward Pb^2+^, while the responses to other ions were effectively suppressed. These results underscore the substantial potential of this method for enhancing the sensor’s anti-interference capability and mitigating cross-responding.

### 2.8. Temperature Response Characteristics

**Figure 11:**
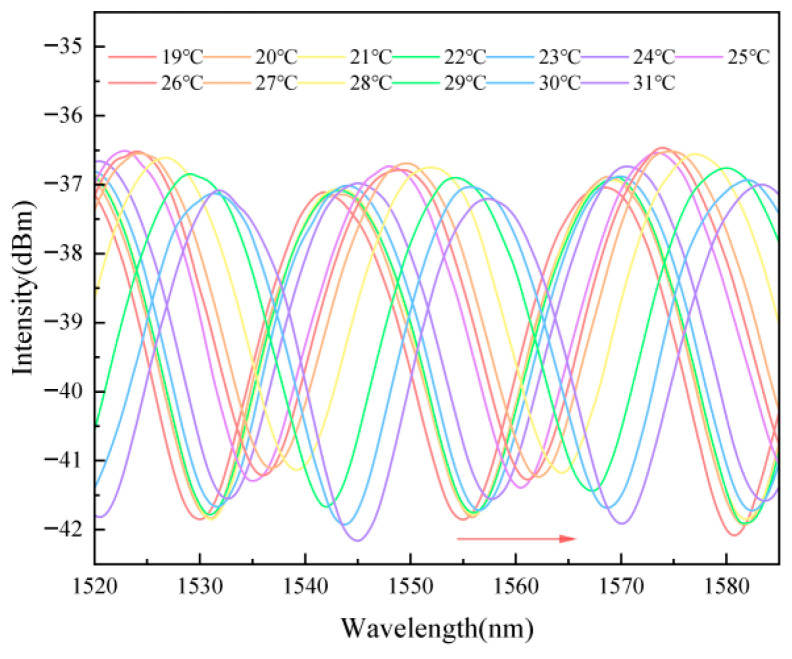
Response spectra of the sensor after stabilization at different temperatures; a redshift occurs with increasing temperature.

For further investigating the sensor’s responsibility, temperature response was also tested. In experiments, the sensor was placed in deionized water, and the temperature was elevated from 19 °C to 31 °C at 1 °C intervals. Figure 11 is the response spectrum figure, which shows that as the temperature increases, the interference valleys in the reflection spectrum experience a redshift. This is due to the thermal expansion of the hydrogel caused by temperature changes [40], which alters the thickness and refractive index of the sensitive film.

It is worth noting that, in contrast to the redshift (i.e., a shift toward longer wavelengths) caused by temperature increase, the sensor responding to metal ions consistently showed blueshift (i.e., a shift toward shorter wavelengths). This opposite spectral behavior serves as a critical distinguishing feature, which not only provides a clear optical signature for identifying ion-response mechanisms but also facilitates effective discrimination between target ion signals and temperature-induced crosstalk in complex environments.

**Figure 12:**
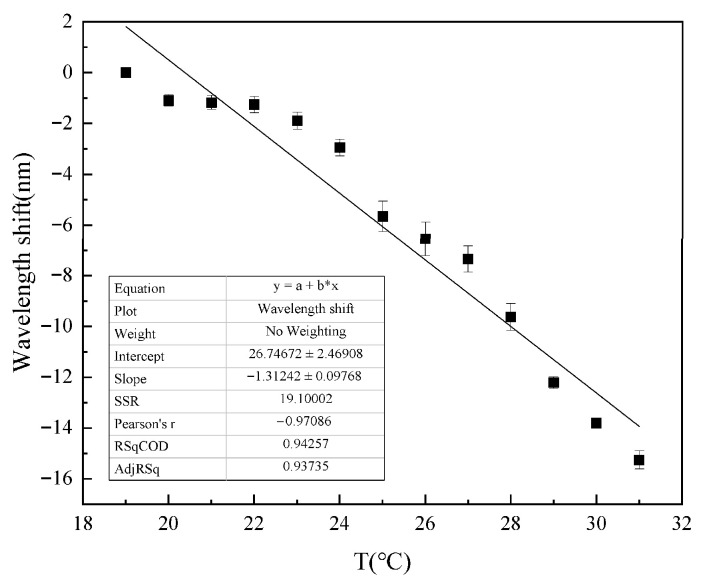
Temperature response curve. The temperature response sensitivity of the sensor is −1.31242 nm/°C with a linearity of 0.942.

As shown in Figure 12, the measured temperature sensitivity of the spectrum is −1.31242 nm/°C, with a linearity of 0.942. To eliminate environmental temperature interference, fiber Bragg gratings (FBGs) can be used in series [41,42,43]. In Pb^2+^ responding experiments, to avoid cross-response caused by temperature, we maintain a constant temperature in the measurement experiments with a temperature deviation within 0.1 °C.

### 2.9. Preliminary Investigation on Sensor Performance Under Dry–Wet Cycling

To evaluate the sensor’s response to dry–wet cycles, a preliminary experimental study was conducted. The fabricated sensor was immersed in deionized water, and the Pb^2+^ concentration was adjusted to 0.5 mg/L. The initial wavelength shift response was recorded after the signal stabilized. The sensor was then carefully removed from the solution, air-dried under ambient conditions, and re-immersed into the same solution, after which the spectral shift response was recorded again.

**Figure 13:**
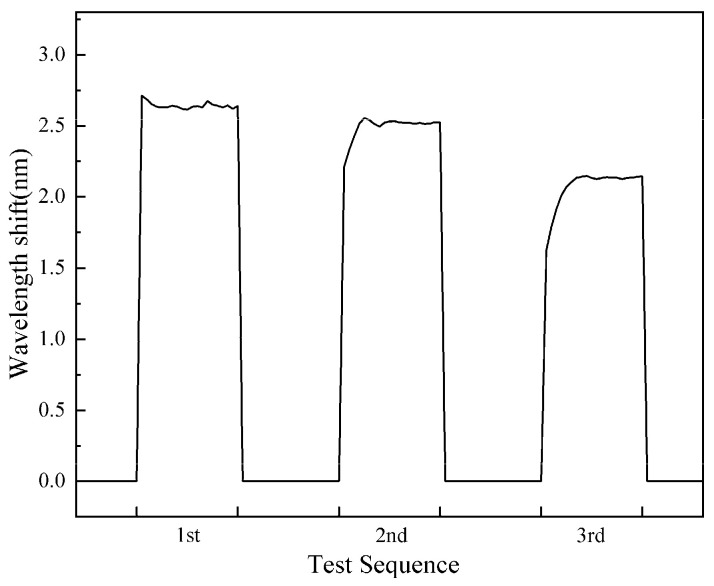
Wavelength shift recovery after dry–wet cycling across repeated experiments.

The responses of the wavelength shift in three dry–wet circles are illustrated in Figure 13. The first cycle (1st) represents the initial spectral shift, while the second (2nd) and third (3rd) cycles correspond to the spectral responses, respectively, followed by re-immersion. The wavelength shift values of three dry–wet circles are 2.615 nm, 2.521 nm, and 2.135 nm.

These results indicate that the sensor retains high sensitivity after dry–wet cycles. However, a decline in sensitivity was also observed. This phenomenon could be attributed to two primary factors: (1) inadequate protection of the fiber optic probe during experimental operation and (2) insufficient mechanical robustness of the sensing material. The former may result from suboptimal fixation of the probe within the experimental setup, while the latter likely stems from limitations in the material’s tensile strength and flexibility. In order to achieve better response characteristics to dry–wet cycles, we will improve the experimental device and the mechanical properties of sensitive materials in future work.

### 2.10. Comparison with Reported Results

Some reported Pb^2+^ concentration optical fiber sensors and our work are listed in Table 2 for comparison. Compared with the optical absorption sensor in reference [44], our sensor has higher sensitivity. Compared with the fiber Bragg grating sensor in reference [45], this sensor does not need to use precious metals such as gold and does not need complex manufacturing processes such as polishing and etching. Moreover, with low cost and high mechanical strength, it also has a larger detection range and higher sensitivity. Compared with the SPR optical fiber sensor in reference [46], the sensor has a simpler manufacturing process, lower cost, and higher sensitivity. Compared with the MZI optical fiber sensor in references [24,47], this sensor has higher sensitivity and does not need to splice optical fibers. Compared with the FPI optical fiber sensor based on chitosan in reference [23], the interpenetrating polymer (IPN) hydrogel proposed in this paper has better Pb^2+^ adsorption and swelling properties, so it has higher response sensitivity to Pb^2+^ concentration change. Moreover, the larger end face of the ceramic insert can make the coated hydrogel film have a larger radius of curvature, which greatly reduces the influence of reflected spectrum quality.

## 3. Conclusions

An FPI fiber sensor was proposed and demonstrated for detecting Pb^2+^ concentration in solutions. By integrating fiber sensing technology with the adsorption characteristics of IPN hydrogels for heavy metal ions, this sensor effectively detects Pb^2+^ concentration, with good sensitivity, linearity, repeatability, selectivity, and stability. The sensor exhibits a high sensitivity of 5.0743 nm/ppm within the linear range of 0–1 ppm, with a linearity of 0.994. Comprehensive performance investigations were conducted, including the influence of the HEMA/AM weight ratio on sensitivity, sensor repeatability, selectivity, dynamic response, and temperature response. Compared with existing detection methods, this sensor offers advantages such as good response characteristics, easy fabrication, and low cost, which shows significant potential for applications in industrial wastewater treatment and water environmental pollution monitoring.

This paper investigated and demonstrated some response performance of the sensor. The effects of different characteristic parameters, such as the manufacturing process, cavity length, and solution pH value, require further in-depth research. Additionally, several important aspects will be emphasized in our future work: Firstly, while the current study focused on the concentration range of 0–1 ppm, future work will extend to systematic investigations of the sensor’s response across broader concentration ranges. Secondly, we will focus on evaluating the sensor’s selective adsorption performance and anti-interference capability in mixed-ion systems and real water samples to fully validate its potential for practical environmental monitoring applications. Thirdly, in response to the current maximum wavelength shift of approximately 5 nm at 1 ppm Pb^2+^, subsequent efforts will systematically optimize key parameters such as hydrogel thickness, crosslinking density, and F-P cavity length to further enhance sensor sensitivity. Fourth, the mechanical stability and performance retention of the sensor under dry–wet cycling conditions will be systematically investigated. Key research efforts will focus on enhancing the adhesion of the hydrogel–substrate interface, improving resistance to dehydration-induced stress, and optimizing the experimental system and operational protocols. Future studies will continue to optimize and improve sensor performance.

## 4. Materials and Methods

### 4.1. Materials

In experiments, single-mode fiber (SMF, 8.3/125 μm) was from Wuhan Changfei Fiber Optics and Cable Co., Ltd. (Wuhan, China); ceramic ferrules were from Shenzhen Hailixing Optoelectronics Co., Ltd. (Shenzhen, China); acrylamide (AM), 2-hydroxyethyl methacrylate (HEMA, 96%), N,N′-methylene bisacrylamide (MBA), and lead chloride (PbCl_2_) were from Shanghai Meilun Biochemical Co., Ltd. (Shanghai, China); and sodium alginate (SA) and ammonium persulfate (APS) were from Shanghai Aladdin Biochemical Co., Ltd. (Shanghai, China).

### 4.2. Structure and Principle

**Figure 14:**
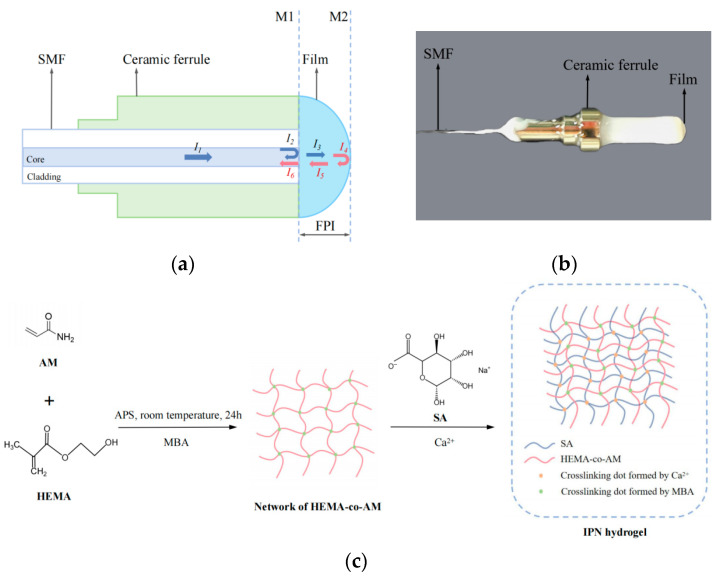
(**a**) Schematic diagram of the sensor, consisting of a single-mode fiber, a ceramic ferrule, and an IPN hydrogel film. (**b**) Photograph of the sensor. (**c**) Synthesis process of the IPN hydrogel, fabricated by a stepwise crosslinking method.

Figure 14 illustrates the structure of the sensor, which consists of a single-mode fiber (SMF), a ceramic ferrule, and an IPN hydrogel film. A schematic representation of the sensor is provided in Figure 14a, accompanied by a photograph of the actual device in Figure 14b. The endface of the single-mode fiber is aligned with the endface of the ceramic ferrule, which has been roughened to achieve better adhesion of the hydrogel. The single-mode fiber tip and the hydrogel film jointly define a Fabry–Perot (F-P) interferometric cavity.

The light transmission process in the sensor is shown in Figure 14a. When a light beam with intensity *I*_1_ is transmitted from one end of the single-mode fiber to the M_1_ interface (the interface between the single-mode fiber and the IPN hydrogel film), Fresnel reflection occurs due to different refractive indices on both sides of the M1 interface. A portion of the light with intensity *I*_2_ is reflected into the single-mode fiber, while the remaining transmitted light with intensity *I*_3_ continues to enter the IPN hydrogel film. When this transmitted light reaches the M_2_ interface (the interface between the IPN hydrogel film and the solution), a portion of the light with intensity *I*_4_ is reflected and transmitted to the M_1_ interface, a portion of the transmitted light with intensity *I*_6_ re-enters the core of the single-mode fiber, and a small remaining portion of light undergoes secondary reflection within the FP cavity with very low reflected intensity. Therefore, the reflected light *I*_2_ and the transmitted light *I*_6_ at the M_1_ interface superimpose to produce interference [48], thereby forming F-P interference.

According to light interference theory, the total intensity of the interfering light can be expressed as follows [49,50,51]:

(1) $$I=I_{2}+I_{6}+2\sqrt{I_{2}I_{6}}cos\left(\frac{4\pi nL}{\lambda }+\varphi_{0}\right),$$

where *φ*_0_ represents the initial phase; *λ* is the wavelength of the light source; and *n* and *L* are the effective refractive index and cavity length of the F-P cavity, respectively. When the phase difference between two lights is $∆\varphi =\frac{4\pi nL}{\lambda_{m}}=2m\pi$ (where *m* is a positive integer), interference peaks appear in the reflection spectrum; the peak wavelength is expressed as follows:

(2) $$\lambda_{m}=\frac{2nL}{m},$$

It can be derived from Equations (1) and (2) that the wavelength spacing between two adjacent peaks, which is the Free Spectrum Range (FSR), is given by the following expression:

(3) $$FSR=|\lambda_{m}-\lambda_{m+1}|=\frac{\lambda_{m}\lambda_{m+1}}{2nL},$$

When the IPN hydrogel material is influenced by external Pb^2+^ concentration, both its refractive index and volume (i.e., effective cavity length) undergo changes. These changes further lead to alterations in the optical path difference of interference, ultimately causing an interference spectrum shift. By measuring the changes in the interference spectrum, the concentration of Pb^2+^ in the solution can be measured. The Pb^2+^ response sensitivity of peak wavelength drift in the interference spectrum can be expressed as follows:

(4) $$S_{\lambda }=\frac{\Delta \lambda }{\Delta c}=\lambda \left(\frac{1}{n}\frac{dn}{dc}+\frac{1}{L}\frac{dL}{dc}\right),$$

where $S_{\lambda }$ represents the response sensitivity of the sensor to Pb^2+^; Δλ represents the peak wavelength drift of the interference spectrum; and Δc represents the change in Pb^2+^ concentration.

We use N,N-methylene bisacrylamide (MBA) to crosslink 2-hydroxyethyl methacrylate (HEMA) and acrylamide (AM) to form P(HEMA-co-AM) as the first network. Then, we prepare P(HEMA-co-AM)/SA IPN hydrogel films by Ca^2+^ to crosslink SA as the second network. Figure 14c shows the synthesis process. AM and HEMA are well-known functional monomers [52,53,54,55], and the hydrogel network formed by their crosslinking exhibits good affinity and selectivity for lead ions. SA hydrogels contain many hydroxyl (-OH) and carboxyl (-COOH) groups in their molecular structure, which can undergo ion exchange reactions with heavy metal ions [56,57], thereby achieving adsorption of heavy metal ions. The dual network (DN) hydrogels synthesized by interpenetrating polymer (IPN) technology [58] exhibit better mechanical properties, swelling properties, and lead ion adsorption capacity.

### 4.3. Sensor Fabrication and Test Solution Preparation

The sensor fabrication process is as follows. Place the end-cut single-mode fiber into a ceramic sleeve with a roughened end face, adjust until the fiber end face is aligned with the circular end face of the ceramic sleeve, then bond the fiber to the tail end of the ceramic sleeve with adhesive to form the sensor’s initial structure. The distal end of the single-mode fiber was connected to a Micron Optics SM125 fiber sensor analyzer. To control the thickness of the hydrogel film, take a small amount of un-crosslinked P(HEMA-co-AM)/SA hydrogel solution and spread it on a glass slide. Then, we use the sensor’s initial structure to dip a little hydrogel and form a hydrogel film on the end face, constructing a Fabry–Perot (F-P) cavity. We optimized the cavity alignment until the SM125 sensor displayed a stable and clear interference pattern. Then, we place it in a 2.5% calcium chloride solution for crosslinking to form a P(HEMA-co-AM)/SA IPN hydrogel film. After crosslinking for a period until curing is complete, we immerse it in deionized water for 24 h to elute all non-reactive materials until the interference spectrum stabilizes. The photo of the completed sensor is shown in Figure 14b.

The P(HEMA-co-AM)/SA IPN hydrogel film was prepared by the stepwise crosslinking method. A homogeneous mixture containing deionized water, AM, HEMA, PVA, APS, and MBA was prepared. The mixture was heated to 50 °C and stirred for 1 h to achieve complete uniformity, followed by ultrasonic treatment for 30 min. Subsequently, the mixture was allowed to stand at room temperature for 24 h to facilitate complete free radical polymerization, enabling the P(HEMA-co-AM) copolymer to penetrate sodium alginate (SA) and form a hydrogel solution. The hydrogel was uniformly coated onto the sensor end face, and the sensor was immersed in a 2.5% calcium chloride (CaCl_2_) solution to ensure sufficient crosslinking, resulting in the formation of a P(HEMA-co-AM)/SA IPN hydrogel film.

### 4.4. Experimental System

**Figure 15:**
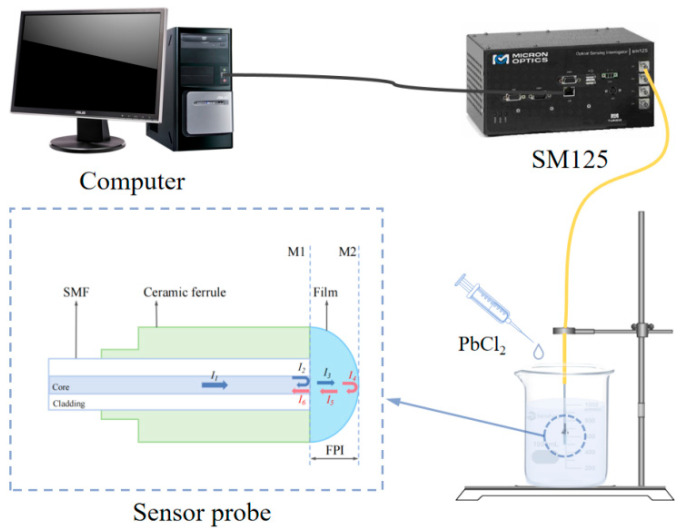
Diagram of the experimental system, comprising a computer, an optical spectrum analyzer, a test solution, and a sensing probe.

As shown in Figure 15, a fiber Bragg grating interrogator (Micron Optics SM125) was employed as the light source and spectral acquisition unit. This instrument provides 18 mW output power, 2 Hz scanning frequency, and 1510–1590 nm output wavelength, with 1 pm wavelength accuracy and stability. The optical signal is transmitted through an optical fiber to an F-P sensing probe, where interference occurs. The resultant beam is then reflected along the same path to the interrogator, which captures and records the interference spectrum. During the experiment, the sensor was immersed in deionized water with an initial pH of 6.2 (±0.2). By incrementally adding a PbCl_2_ solution, the concentration of Pb^2+^ ions was systematically increased. The sensor’s response characteristics were evaluated by examining correlations between spectral shifts and ion concentration variations.

## Figures and Tables

**Table 1:** Chemical composition of P(HEMA-co-AM)/SA IPN hydrogel film.

Sample Codes	HEMA (g)	AM (g)	SA (g)	H_2_O (mL)
10% HEMA	0.1	0.9	1	48
30% HEMA	0.31	0.7	1	48
50% HEMA	0.52	0.5	1	48
70% HEMA	0.73	0.3	1	48
90% HEMA	0.94	0.1	1	48

**Table 2:** Comparison with other optical fiber Pb^2+^ concentration sensors.

Sensitive Material	Sensing Probe	Mechanism	Detection Range (ppm)	Sensitivity	Cost	Manufacturing Difficulty	Refs.
Chitosan	SMF	Optical absorbance	0–70	0.044 dBm/ppm	Low	Easy	[44]
AuNPs-maleic acid	Etched-FBG	FBG	0–0.02	2.703 × 10^−6^ nm/ppm	High	Difficult	[45]
1,1-Mercaptoundecanoic acid	MMF coated by AuNPs	LSPR	166–2 × 10^4^	1.35 × 10^−3^ nm/ppm	High	Difficult	[46]
Ionic imprinting chitosan	HCF-TCF-HCF	MZI	0–350	0.01268 nm/ppm	High	Difficult	[47]
P(HEMA-co-AM) hydrogel	NCF-FMF-NCF	MZI	0.04–0.25	3.936 nm/ppm	High	Difficult	[24]
Spherically shaped chitosan diaphragm	SMF-Capillary	FPI	0–30	0.091 nm/ppm	Low	Medium	[23]
P(HEMA-co-AM)/SA IPN hydrogel	SMF-Ceramic ferrule	FPI	0–1	5.22118 nm/ppm	Low	Easy	This work

## Data Availability

The data underlying the presented results are not publicly available but may be obtained from the authors upon reasonable request.
